# Stat3 role in the protective effect of FXR Agonist in parenteral nutrition-associated cholestasis

**DOI:** 10.1097/HC9.0000000000000056

**Published:** 2023-02-27

**Authors:** Swati Ghosh, Michael W. Devereaux, Aimee L. Anderson, *Karim C. El Kasmi, Ronald J. Sokol

**Affiliations:** 1 Section of Pediatric Gastroenterology, Hepatology and Nutrition, Department of Pediatrics, Pediatric Liver Center, University of Colorado School of Medicine, Aurora, Colorado, USA; 2 Digestive Health Institute, Children’s Hospital Colorado, Aurora, Colorado, USA

## Abstract

Parenteral nutrition (PN) in patients with intestinal failure can lead to cholestasis (PNAC). In a PNAC mouse model, farnesoid X receptor (FXR) agonist (GW4064) treatment alleviated IL-1β-dependent cholestatic liver injury. The objective of this study was to determine whether this hepatic protection of FXR activation is mediated through IL-6-STAT3 signaling.

**Approach and Results:**

Hepatic apoptotic pathways [Fas-associated protein with death domain (*Fas*) mRNA, caspase 8 protein, and cleaved caspase 3] and IL-6-STAT3 signaling, and expression of its downstream effectors *Socs1/3* were all upregulated in the mouse PNAC model (dextran sulfate sodium enterally × 4 d followed by total PN for 14 d). *Il1r* ^*−/−*^ mice were protected from PNAC in conjunction with suppression of the FAS pathway. GW4064 treatment in the PNAC mouse increased hepatic FXR binding to the *Stat3* promoter, further increased STAT3 phosphorylation and upregulated *Socs1* and *Socs3* mRNA, and prevented cholestasis. In HepG2 cells and primary mouse hepatocytes, IL-1β induced *IL-6* mRNA and protein, which were suppressed by GW4064. In IL-1β or phytosterols treated HepG2 and Huh7 cells, siRNA knockdown of *STAT3* significantly reduced GW4064-upregulated transcription of hepatoprotective nuclear receptor subfamily 0, group B, member 2 (*NR0B2*) and *ABCG8.*

**Conclusions:**

STAT3 signaling mediated in part the protective effects of GW4064 in the PNAC mouse, and in HepG2 cells and hepatocytes exposed to either IL-1β or phytosterols, 2 factors critical in PNAC pathogenesis. These data demonstrate that FXR agonists may mediate hepatoprotective effects in cholestasis by inducing STAT3 signaling.

## INTRODUCTION

Parenteral nutrition-associated liver disease is an important complication of long-term PN administration to children and adults with intestinal failure. Parenteral nutrition-associated liver disease, more recently called intestinal failure-associated liver disease, is characterized primarily by cholestasis (PNAC) in children, and is the leading indication for intestine or multivisceral transplantation in these patients^1^–^3^ Earlier, we reported in a mouse model of PNAC that macrophage-derived proinflammatory cytokines IL-1β and TNF-α play important roles in the pathogenesis of PNAC.^4^–^6^ However, the effects of the increased hepatic expression of *IL-6* in PNAC^5^–^10^ are uncertain, as IL-6 may both promote inflammation and development of hepatic fibrosis,^11^,^12^ as well as mediate protection against liver injury.^13^,^14^ IL-6 acts through the activation of STAT3 pathways,^15^–^17^ which may inhibit or promote apoptosis and cell proliferation depending on the tissue and the intracellular site of action.^18^ In the liver, IL-6-STAT3 signaling protects against ischemia and reperfusion injury, toxin-mediated injury, and Fas-associated protein with death domain (FAS)–activated cell death.^19^–^22^ Apoptosis was one of the histologic hallmarks in the PNAC mouse liver.^4^,^23^ The proinflammatory IL-1β regulates FAS-mediated apoptosis at the transcriptional and posttranscriptional levels.^24^,^25^ FAS also plays a crucial role in immune regulation by promoting apoptosis through caspase 8 (Caspase 8) activation.^24^,^26^–^29^ Importantly, mice that are deficient in IL-6-STAT3 signaling have reduced hepatic expression of the antiapoptotic mediators, flice-like inhibitory protein (FLIP), B-cell lymphoma 2 (Bcl-2), and B-cell lymphoma-extra large (Bcl-xL), which are normally induced in the liver by IL-6.^13^,^20^,^30^ Thus, there are several biological interactions between IL-1β, FAS, and IL-6-STAT3 activation pathways that may play important roles in PNAC liver injury.

The nuclear receptor FXR (*Nr1h4*) is a bile acid–activated transcription factor,^31^–^34^ which is a key regulator of hepatocyte protective responses to cholestasis.^7^,^35^ Thus, FXR agonists have been used as treatment for human cholestatic liver diseases; however, the mechanisms by which these steroidal compounds elicit liver protection are poorly understood. We have recently reported that the FXR agonist GW4064 prevented PNAC in a mouse model that combines intestinal injury and increased intestinal permeability by dextran sulfate sodium (DSS) with i.v. administration of total PN (DSS-PN mice). The aim of the present study was to determine the role of the STAT3 signaling pathway and its interaction with FXR gene regulation in the PNAC mouse model.

## EXPERIMENTAL PROCEDURES

### PNAC mouse model

All animal experiments were approved by the Institutional Animal Care and Use Committee of the University of Colorado Anschutz Medical Campus and all animals were treated humanely during the experimental techniques. All research was conducted in accordance with both the Declarations of Helsinki and Istanbul.

For the PNAC mouse model,^4^,^36^ C57BL/6 wild-type (WT) or *Il1r* ^*−/−*^ adult male mice (8 wk old, 22–25 g body weight) (Jackson Laboratories, Bar Harbor, ME) were treated sequentially with DSS in drinking water for 4 days, followed by PN for 14 days as reported^36^ and described in Supplementary Experimental Procedures http://links.lww.com/HC9/A133. A subset of DSS-PN mice received the FXR agonist GW4064 (Tocris Bioscience, USA) i.v. 30 mg/kg, as reported.^7^,^36^ Another group of WT mice (8-wk old, 22–25 g body weight) (Jackson Laboratories, Bar Harbor, ME) were administrated i.p. injections of either 2.5 mg/kg/bw *E. Coli* lipopolysaccharides (LPS; from *Escherichia coli* 0111:B4; Sigma Aldrich, St. Louis, MO, USA) or 200ng recombinant mouse IL-1β (BD Biosciences, San Jose, CA) and 30 mg/kg/bw GW4064, as described in Supplementary Experimental Procedures http://links.lww.com/HC9/A133.^7^,^36^

### RNAi transfection and *in vitro* IL-1β incubation experiments

HepG2 and Huh7 cells (ATCC, Manassas, VA) in culture were treated with 100 nM siRNA-STAT3 (cat: L-003544-00-0005, Dharmacon, Lafayette, CO) or a nontargeting siRNA (siRNA-NTC, cat: D-001810-10-05, Dharmacon) in the presence of Dharmafect transfection reagent for 24 hour. The next day, cells were treated with either combinations of 10 ng/mL IL-1β, 5 μM GW4064 or 10 μM stigmasterol acetate (Stig-Ac) and 10 μM sitosterol acetate (Sit-Ac) (Steraloids, Newport, RI) for 20 hours in DMEM-serum media with transfection mixture, as described.^6^ In a separate set of experiment, *in vitro* IL-1β incubation was carried out in HepG2 and in Huh7 cells in culture by treating cells with 10 ng IL-1β (BD Biosciences, San Jose, CA) for 4 hours followed ±5 μM GW4064 overnight in DMEM media.

### RNA isolation and quantitative gene expression analysis

Liver or cellular RNA was extracted using TRIzol (Invitrogen, Carlsbad, CA), according to manufacturer’s instructions. All RNA samples were used for real time quantitative PCR, as described.^36^ All TaqMan probes used are listed in Supplemental Table 1 (http://links.lww.com/HC9/A133).

### Antibodies and Immunoblot analysis

Total cell lysates of HepG2 and cultured mouse hepatocytes cells and liver homogenates were extracted using M-PER Extraction Reagents (Thermo Fisher Scientific, Waltham, MA), as described.^6^ The antibodies used in this study are listed in Supplemental Table 2 (http://links.lww.com/HC9/A133).

### IL-6 ELISA

To detect human IL-6 protein in cell culture media and cell lysates, the human IL-6 Legend Max ELISA Kit (Cat No: 430507; Bio Legend, San Diego, CA) and mouse IL-6 ELISA kit (Cat No: BMS603-2;Thermo Fisher Scientific, Waltham, MA) were used, according to the manufacturer’s instructions.

### Chromatin immunoprecipitation

Chromatin immunoprecipitation (ChIP) assay was performed on liver samples using EZ ChIP/Magna ChIP G Kit from EMD/Millipore (Billerica, MA), according to the manufacturer’s instructions. ChIP was done using specific antibody for FXR amplification of promoter sequences from the *Stat3*, *Abcg8*, nuclear receptor subfamily 0, group B, member 2 (*Nr0b2*), *and Socs3* using specific primer sets (Qiagen, USA) and subsequent quantitative PCR.

### Flow cytometry

Intrahepatic mononuclear cells and mouse primary hepatocytes were isolated from the liver as described previously^36^, washed, pelleted, and resuspended in staining buffer (Thermo Fisher Scientific, Waltham, MA) and stained with specific antibodies, as described.^36^ Primary antibodies that were used targeted F4/80, CD178 and CD95 (Thermo Fisher Scientific, Waltham, MA). Flow-assisted cell analysis was conducted at the University of Colorado Cancer Center Flow Cytometry Core Facility and were performed on Yeti flow cytometry device (Bio-rad) and data were analyzed by Kaluza software (Beckman Coulter, Brea, CA).

### Statistical analysis

Quantitative PCR assays were determined in triplicate for each mouse and the average from each mouse was used to calculate the mean±SE of mean for each treatment group. For cell culture experiments, gene expression was determined in triplicate, and results from 3 representative experiments are shown. To ensure rigor, animals were randomly allocated to treatment groups and all samples were coded and were analyzed blinded to the treatment group. The number of mice in each experimental group was 3–7 and is provided in the text, figures, and/or legends. One-way ANOVA and Tukey correction for multiple comparisons were used to determine statistical significance for multiple group comparisons. The level of significance between 2 groups was determined by Student 2-tailed unpaired *t* test. *p*-value <0.05 was considered statistically significant. The statistical analysis was done in PRISM Graph Pad software (La Jolla, CA).

## RESULTS

### FAS-mediated apoptosis is activated during PNAC

**FIGURE 1:**
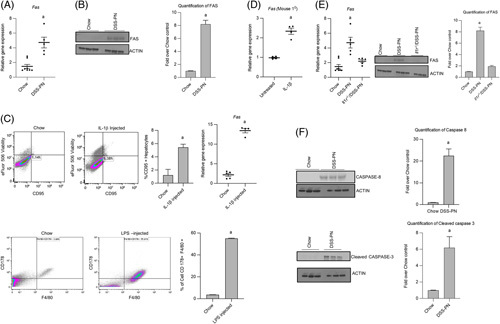
FAS-mediated apoptosis is activated during parenteral nutrition-associated cholestasis. (A) Quantitative PCR (qPCR) analysis of hepatic *Fas* mRNA from Chow and DSS-PN mice. *Hprt1* was used as a reference control gene and expressed relative to Chow controls. n for chow=8, DSS-PN=5. (B) Immunoblot of FAS from liver homogenate of Chow and DSS-PN mice. Quantification of integrated density values (IDV) of FAS immunoblot using actin as reference control relative to Chow control. n for chow=3, DSS-PN=3. (C) Flow cytometry (n for chow=3, DSS-PN n=3) and qPCR (n for chow=5, DSS-PN n=4) of Fas/CD95 positive hepatocytes from Chow and recombinant IL-1β-injected wild-type (WT) mice or F4/80 and CD178 positive macrophages (n for chow=3, DSS-PN n=3 ) from Chow and LPS injected WT mice. (D) qPCR of *Fas* in mouse hepatocytes that were incubated with IL-1β overnight. n for chow=4, DSS-PN n=4. (E) qPCR and immunoblot analysis of hepatic *Fas* from WT/Chow, WT/DSS-PN, and *Il1r* ^*−/−*^ */*DSS-PN mice. n for chow=8, DSS-PN n=5, *Il1r* ^*−/−*^ */*DSS-PN n=5 . IDV of immunoblot of FAS normalized to actin as reference control relative to Chow control. n for chow=3, DSS-PN n=3, *Il1r* ^*−/−*^ */*DSS-PN n=3 **(F)** Immunoblot of liver protein of caspase 8 and cleaved caspase 3 in Chow and DSS-PN mice. n for chow=3, DSS-PN n=3. IDV of immunoblots of caspase 8 and cleaved caspase 3 normalized to actin and relative to Chow control. Data indicate the mean±SEM. ^a^ *p<*0.05 versus control group or all other groups, (A–D, F) by 2-tailed, unpaired Student’s *t* test and (E) 1-way ANOVA. Abbreviations: DSS, dextran sulfate sodium; DSS-PN, dextran sulfate sodium-parenteral nutrition; Fas, Fas-associated protein with death domain; LPS; lipopolysaccharides.

Apoptosis of hepatocytes is mediated in cholestasis through the cell surface molecule FAS.^29^,^37^–^39^ Ligand binding to the FAS receptor on hepatocytes induces caspase 8 activation that initiates the FAS-mediated pathway of apoptotic cell death.^40^ To determine whether the evidence of FAS-mediated apoptosis is present in PNAC, we first measured the hepatic expression of *Fas* in 14 days DSS-PN and chow mice and showed increased *Fas* mRNA and FAS protein were associated with cholestasis in DSS-PN mice (Figure 1A, B). Next, to determine whether IL-1β might be responsible for the activation of *Fas* in this model, we injected WT mice with 200 ng IL-1β by i.p.; 3 hours later, hepatocytes were isolated and stained them with CD95/FAS antibody followed by flow cytometry and performed quantitative PCR. The results showed that IL-1β-treated mice had increased percentage of FAS-positive hepatocytes compared with control mice and that *Fas* mRNA expression was increased in isolated hepatocytes (Figure 1C). We next sought to determine whether LPS, which we have shown is absorbed from the hyperpermeable intestine in the PNAC mouse and is associated with increased lL-1β production by hepatic macrophages,^7^,^36^ is capable of upregulating FAS ligand expression in intrahepatic macrophages. We measured FAS ligand (*Fasl*; CD178) expression by flow cytometry in intrahepatic mononuclear cells isolated from LPS i.p.-injected WT mice and found elevated CD178 expression in F4/80-positive macrophages compared with WT mice (Figure 1C). Next, we analyzed *Fasl* expression in intrahepatic mononuclear cell of DSS-PN and chow mice and similarly observed the upregulation of *Fasl* mRNA *in vivo* during PNAC (Supplemental Figure 1A, http://links.lww.com/HC9/A128). Finally, we treated cultured primary mouse hepatocytes with IL-1β overnight and found increased *Fas* mRNA expression (Figure 1D). Using murine models, we have previously reported that absence of the IL-1 receptor (IL-1 receptor-deficient mice; *Il1r* ^−/−^) or treatment with the IL-1r antagonist anakinra prevented PNAC.^36^ To further examine the regulation of IL-1β on *Fas* expression during PNAC, *Il1r* ^−*/*−^ mice were subjected to DSS-PN treatment as in WT mice and showed protection from PNAC as described in our previous study.^36^ Hepatic mRNA and protein were used from mouse livers obtained in these prior experiments for the current study. Hepatic expression of *Fas* mRNA and FAS protein were significantly decreased in *Il1r* ^−*/*−^/DSS-PN mice when compared with WT/DSS-PN mice (Figure 1E). Taken together, these data support the roles of intestinally derived LPS in upregulating *Fasl* on intrahepatic macrophages and of activated hepatic macrophage-derived IL-1β signaling in upregulating hepatocyte *Fas/*FAS expression in the PNAC mouse model.

We next sought to determine whether the upregulated FAS and *Fasl* expression was associated with activation of apoptosis in the PNAC mouse.^41^ We performed immunoblotting for caspase 8 and cleaved caspase 3, 2 downstream apoptosis mediators after FAS activation, and show increased protein expression of both in DSS-PN mouse liver and CASP3 in cultured mouse hepatocytes (Figure 1F; Supplemental Figure 1B, http://links.lww.com/HC9/A128).

### IL-1β promotes STAT3 signaling during PNAC

**FIGURE 2:**
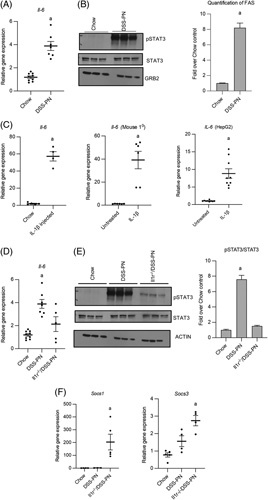
Liver injury initiates STAT3 signaling during parenteral nutrition-associated cholestasis. (A) Quantitative PCR (qPCR) of hepatic *Il-6* from Chow and DSS-PN mice. mRNA expression was determined after normalization to *Hprt1* as reference control gene and expressed relative to results obtained from Chow controls. n for chow=9, DSS-PN n=7 (B) Immunoblot of pSTAT3 in liver homogenates from Chow and DSS-PN mice. Integrated density value (IDV) of immunoblot of pSTAT3 normalized total STAT3 and GRB2 and expressed relative to Chow control. n for chow=3, DSS-PN n=3. (C) qPCR of *Il-6* in purified mouse hepatocytes (n for chow=5, DSS-PN n=4) from mouse 3 hours after Il-1β injection, and primary mouse hepatocytes (n for chow=6, DSS-PN=6) and HepG2 cells (n=3) incubated with IL-1β overnight. *Hprt1* used as reference control. (D) qPCR of hepatic *Il-6* from Chow, DSS-PN and *Il1r* ^*−/−*^ */*DSS-PN mice. *Hprt1* was used as reference control gene. n for chow=9, DSS-PN n=7, *Il1r* ^*−/−*^ */*DSS-PN n=5 (E) Immunoblot of pSTAT3 in liver homogenates from Chow, WT/DSS-PN and *Il1r* ^*−*/*−*^/DSS-PN mice. IDV of immunoblot of pSTAT3 normalized to STAT3 and GRB2 and expressed relative to Chow control. n for chow=3, DSS-PN n=3, *Il1r* ^*−/−*^ */*DSS-PN n=3. (F) qPCR of hepatic *Socs1* (n for chow=4, DSS-PN n=3, *Il1r* ^*−/−*^ */*DSS-PN n=5) and *Socs3* (n for chow=5, DSS-PN n=4, *Il1r* ^*−/−*^ */*DSS-PN n=4) from Chow, DSS-PN and *Il1r* ^*−/−*^ */*DSS-PN mice. *Hprt1* was used as reference control. Data indicate the mean±SEM. ^a^ *p<*0.05 versus control group or all other groups, by 2-tailed, unpaired Student’s *t* test (A–C) and 1-way ANOVA (D–F). Abbreviations: DSS-PN, dextran sulfate sodium-parenteral nutrition; FXR, farnesoid X receptor; STAT3, signal transducer and activator of transcription 3.

Activation of the IL-6-STAT3 pathway has been shown to provide liver protection by regulating gene expression (eg, *SAA2* and *SOCS*) in hepatocytes.^19^ To evaluate the IL-6-STAT3 pathway in PNAC, we next measured the expression of *IL-6* and STAT3 phosphorylation in mouse liver and show upregulated mRNA expression of *Il-6* and increased pSTAT3 in DSS-PN mice compared with WT mice (Figure 2A, B). We previously reported that IL-1β-mediated suppression of FXR signaling was a key factor in the pathogenesis of cholestasis in the PNAC mouse model.^6^,^7^,^36^ To determine the role of IL-1β in activation of the IL-6-STAT3 pathway in the liver, we treated WT mice with i.p. injection of IL-1β and exposed cultured primary mouse hepatocytes and HepG2 cells to IL-1β overnight and measured *Il-6* mRNA expression. In all these experiments, we observed increased expression of *Il-6/IL-6* mRNA after IL-1β treatment and increased IL-6 protein and pSTAT3 in HepG2 cell incubations with IL-1β (Figure 2C, Supplemental Figure 2A–C, http://links.lww.com/HC9/A129). We next examined the contribution of IL-1β to the activation in *Il-6* and STAT3 expression *in vivo* in the PNAC mouse. Hepatic mRNA expression of *Il-6* and STAT3 phosphorylation were significantly increased in WT/DSS-PN mice but were markedly lower in *Il1r* ^*−/−*^/DSS-PN mice when compared with the upregulation observed in WT/DSS-PN mice (Figure 2D, E), supporting a role of IL-1 signaling in upregulating *Il-6* expression and promoting STAT3 phosphorylation in the PNAC mouse model. Because SOCS, which is upregulated by STAT3, has been shown to itself inhibit activation of STAT3,^42^ *Socs1/3* expression in *Il1r* ^*−/−*^/DSS-PN mice was compared with WT/DSS-PN mice and showed upregulation of *Socs1/3* in *Il1r* ^*−/−*^/DSS-PN mice (Figure 1F), suggesting that increased *Socs1/3* may play a supplementary inhibitory role in the activation of STAT3 in *Il1r* ^*−/−*^/DSS-PN mice.

### GW4064 promotes STAT3 activation and reduced apoptosis in PNAC

**FIGURE 3:**
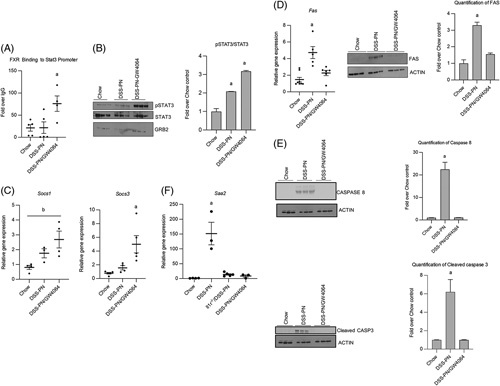
STAT3 activation by GW4064 downregulates apoptosis pathways. (A) chromatin immunoprecipitation assay for FXR binding to the promoter of *Stat3* in liver homogenate from Chow, DSS-PN, and DSS-PN/GW4064 mice. Data presented as fold change over IgG. n for chow=5, DSS-PN n=5, DSS-PN/GW4064 n=4. (B) Immunoblot of pSTAT3 in liver homogenates from Chow, DSS-PN, and DSS-PN/GW4064 mice. Integrated density value (IDV) of immunoblot of pSTAT3 normalized to total STAT3 and GRB2 and expressed relative to Chow control. n for chow=3, DSS-PN n=3, DSS-PN/GW4064 n=3. (C) Quantitative PCR (qPCR) of hepatic *Socs1* (n for chow=4, DSS-PN n=3, DSS-PN/GW4064 n=4) and *Socs3* (n for chow=4, DSS-PN n=4, DSS-PN/GW4064 n=5) from Chow, DSS-PN and DSS-PN/GW4064 mice. *Hprt1* was used reference control gene. (D) qPCR of hepatic *Fas* and immunoblot of FAS in liver homogenates from Chow, DSS-PN, and DSS-PN/GW4064 mice. n for chow=8, DSS-PN n=5, DSS-PN/GW4064 n=6. IDV of the FAS immunoblot and normalized to actin as reference control relative to Chow control. n for chow=3, DSS-PN n=3, DSS-PN/GW4064 n=3. (E) Immunoblot of Caspase 8 and cleaved-Caspase 3 in liver from Chow, DSS-PN and DSS-PN/GW4064 mice. IDV of the Caspase 8 and Cleaved Caspase 3 immunoblot and normalized to actin as reference control relative to Chow control. n for chow=3, DSS-PN n=3, DSS-PN/GW4064 n=3. (F) qPCR of hepatic *Saa2* from Chow, DSS-PN, and DSS-PN/GW4064 or *Il1r* ^*−/−*^ */*DSS-PN mice. *Hprt1* was used as a reference control and expressed relative to Chow controls. (n for chow=4, DSS-PN n=3, *Il1r* ^*−/−*^ */*DSS-PN n=5, DSS-PN/GW4064 n=3). Data expressed as mean±SEM. ^a^ *p<*0.05 versus all other groups by 1-way ANOVA. ^b^ *p<*0.05 versus Chow control by 1-way ANOVA. Abbreviations: DSS-PN, dextran sulfate sodium-parenteral nutrition; FXR, farnesoid X receptor.

We previously reported that soybean oil–based lipid emulsions in PN i.v. solutions in mice led to increased hepatic macrophage recruitment, cytokine production, and hepatocyte apoptosis, followed by the suppression of FXR signaling, and subsequently the onset of hepatic injury and cholestasis,^6^,^7^,^36^ all of which were prevented by treatment with an i.v. FXR agonist.^7^ On the basis of the results described above, we hypothesized that the FXR agonist-induced hepatic protection in PNAC may have been mediated through activation of IL-6-STAT3 signaling. To examine this hypothesis, we first measured FXR binding to the *Stat3* promoter by ChIP analysis in liver from 14 days DSS-PN mice or DSS-PN mice treated with GW4064 and chow control mice^7^ (mice that were previously reported in El Kasmi and colleagues). FXR binding to the *Stat3* promoter was markedly increased in DSS-PN/GW4064 mouse liver compared with the other groups (Figure 3A). In addition, GW4064 treatment restored the reduced binding of FXR to the promoters of *Abcg8*, *Nr0b2*, and *Socs3* observed in DSS-PN mice (Supplemental Figure 3C, http://links.lww.com/HC9/A130). Because the phosphorylation status of STAT3 indicates its activation, pSTAT3 was determined in liver using immunoblotting; STAT3 phosphorylation in DSS-PN/GW4064 mice was significantly increased compared with both chow and DSS-PN mice (Figure 3B). Moreover, the *Stat3* targets, *Socs1* and *Socs3*, which suppress the IL-6-STAT3 pathway by inhibiting IL-6,^43^ were also upregulated in DSS-PN/GW4064 mice compared with DSS-PN and chow mice (Figure 3C). Furthermore, expression of the acute phase protein apoptosis activator^44^ *Saa2* was strongly upregulated in DSS-PN liver but normalized with GW4064 treatment and in *Il1r* ^*−/−*^ mice exposed to DSS-PN (Figure 3F). These findings demonstrate that the FXR agonist GW4064 promoted increased FXR binding to the *Stat3* promoter, STAT3 protein phosphorylation and activation, and increased expression of STAT3 downstream targets in the PNAC mouse, which were associated with reduced expression of apoptotic activation (*Fas*/FAS, *Saa2*, caspase 8, and cleaved caspase 3) (Figure 3D–F). Further supporting this mechanism of FXR protection, in primary mouse hepatocytes exposed to IL-1β, GW4064 treatment significantly reduced the elevated mRNA expression of both *Saa2* and *Fas* compared with IL-1β exposure alone (Supplemental Figure 3A, B, http://links.lww.com/HC9/A130).

### GW4064 inhibits IL-1β induction of *IL-6* expression in hepatocytes

**FIGURE 4:**
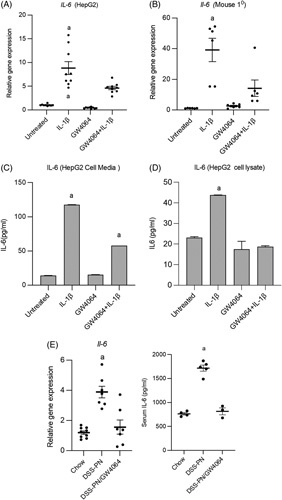
GW4064 inhibits the IL-1β upregulation of IL-6 in hepatocytes and parenteral nutrition-associated cholestasis mice. Cultured cells were incubated with IL-1β for 4 hour, followed by GW4064 overnight after which cells were harvested and *IL-6* quantitative PCR (qPCR) analysis and IL-6 ELISA were performed (A–D). qPCR for *IL-6/Il-6* mRNA from HepG2 cells (n=3) (A) and primary mouse hepatocytes incubated with IL-1β (B) for 4 hours, followed by GW4064 overnight (n=3) (C). IL-6 protein by ELISA from HepG2 Cell media. n=3 (D) and HepG2 cell lysate, n=3 (E). qPCR of hepatic *Il-6* mRNA from Chow, DSS-PN and DSS-PN/GW4064 mice. n for chow=9, DSS-PNn=6, DSS-PN/GW4064 n=7. *Hprt1* was used as reference control gene and results expressed relative to untreated cells or Chow controls. ELISA analysis of IL-6 protein. n for chow=4, DSS-PN n=5, DSS-PN/GW4064 n=3. ^a^=significantly different from all other groups (*p<*0.05). Data indicate the mean±SEM of 3 independent experiments. Abbreviations: DSS-PN, dextran sulfate sodium-parenteral nutrition;

Previously, we have reported an essential role in the PNAC mouse model for altered intestinal permeability and absorption of LPS with activation of hepatic macrophages and release of proinflammatory cytokines, particularly IL-1β.^6^,^7^,^36^ We next sought to determine the effects of GW4064 on IL-1β induction of IL-6 both *in vitro* and *in vivo*. We treated HepG2, Huh7, and primary mouse hepatocytes with IL-1β for 4 hours, followed by GW4064 overnight and found that GW4064 decreased the IL-1β-induced expression of *Il-6* mRNA (Figure 4A, B, F) and IL-6 protein levels in the cell media and in cell lysates (Figure 4C, D). Similarly, GW4064 treatment in DSS/PN mice significantly decreased hepatic mRNA expression of *Il-6* and serum IL-6 protein levels (which were induced in DSS/PN mice) to amounts comparable to chow controls (Figure 4E), indicating that activation of FXR was able to reduce expression of this proinflammatory cytokine to near control levels *in vivo.*

### STAT3 regulates the expression of FXR targets

**FIGURE 5:**
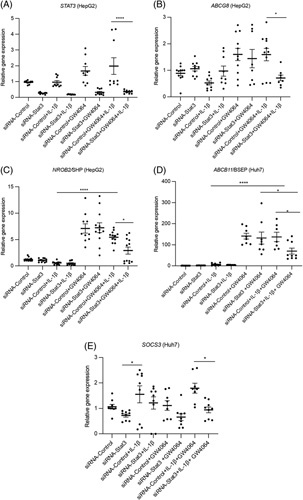
STAT3 inhibition downregulates the expression of farnesoid X receptor target genes. HepG2 or Huh7 cells were transfected with *STAT3* siRNA or nontargeting siRNA control for 24 hours, followed by addition of IL-1β and GW4064 overnight after which cells were harvested and quantitative PCR analysis was performed. n=3. (A) *STAT3*, (B) *ABCG8*, (C) *NR0B2*/SHP, (D) *ABCB11*/BSEP, (E) *SOCS3.* Data indicate the mean±SEM of 3 independent experiments. *p<0.05 by 1-way ANOVA; *p<0.05, **p<0.01, ****p<0.0001. Abbreviations: STAT3, signal transducer and activator of transcription 3.

To further explore the interactions of STAT3 with FXR signaling, we used siRNA-mediated knockdown of STAT3 expression in HepG2 and Huh7 cells (Figure 5A, Supplemental Figure 5A, D, http://links.lww.com/HC9/A131) and observed significant inhibition of expression of FXR target genes *ABCG8 NR0B2, ABCB11*, and *SOCS3* in cells exposed to IL-1β (Figure 5B–E; Supplemental Figure 5B, C, http://links.lww.com/HC9/A131). We previously have shown that GW4064 reverses the suppressive effect of IL-1β on FXR target gene expression in cultured hepatocytes.^6^,^7^,^36^ We now show that siRNA knockdown of STAT3 in HepG2 or Huh7 cells abrogated the ability of GW4064 to reverse the suppressive effects of IL-1β on expression of *ABCG8*, *NR0B2*, *ABCB11*, *and SOCS3*. (Figure 5B–E). Finally, incubation of HepG2 cells with GW4064 or the combination of GW4064 + IL-1β induced STAT3 expression, which was inhibited by STAT3 siRNA knockdown (Figure 5A). Taken together, these findings indicate that FXR regulates its target genes *ABCG58*, *NR0B2*, *ABCB11*, and *SOCS3* in hepatocytes through a STAT3-dependent process during IL-1β exposure.^6^,^7^,^36^

### Downregulation of *STAT3* increases inhibitory effect of phytosterols on FXR target gene expression

**FIGURE 6:**
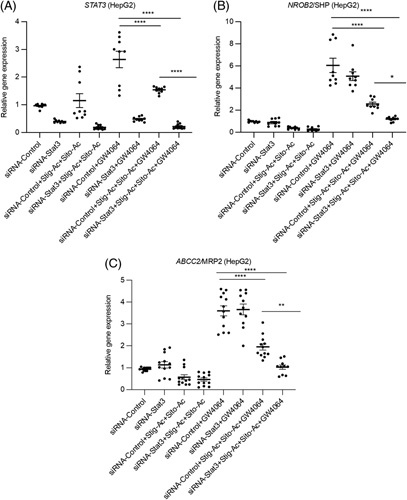
Downregulation of *STAT3* increases inhibitory effect of phytosterols on FXR target gene. HepG2 cells were incubated with *STAT3* for 24 hours, followed by addition of ±GW4064 or ±stig+sito overnight, cells were harvested, and mRNA analyzed. n=3. (A) *STAT3*, (B) *NR0B2*, and (C) *ABCC2/*MRP2. *p<0.05, ***p<0.001, ****p<0.0001. Data indicate the mean±SEM of 3 independent experiments. *p<0.0, by 1-way ANOVA.

Hepatic accumulation of bile acids activates FXR, which induces the expression of canalicular bile transport genes and blocks the transcription of *CYP7A1*, the rate-limiting enzyme of bile acid biosynthesis.^6^ As accumulation of phytosterols (PS) in the liver (from the i.v. lipid emulsion) during PNAC interferes with FXR signaling and bile-transporter expression,^23^ we next examined the interaction between PS, STAT3, and FXR signaling. siRNA knockdown of STAT3 in HepG2 or Huh7 cells significantly reduced *STAT3* mRNA levels (Figure 6A, Supplemental Figure 6A, D, http://links.lww.com/HC9/A132). These cells were then incubated with GW4064, the PS stigmasterol (stig) acetate + sitosterol (sito) acetate, or the combination overnight. The ability of GW4064 to induce transcription of the FXR target genes *NR0B2 and* ATP-binding cassette subfamily C member 2 (*ABCC2*) (Figure 6B, C; Supplemental Figure 6B, C, http://links.lww.com/HC9/A132) was significantly reduced in the presence of stig+ sito and was further reduced with *STAT3* siRNA knockdown. Thus, suppression of *STAT3* was associated with further reduction in the expression of GW4064-induced FXR target genes in cultured cells exposed to PS.

## DISCUSSION

**FIGURE 7:**
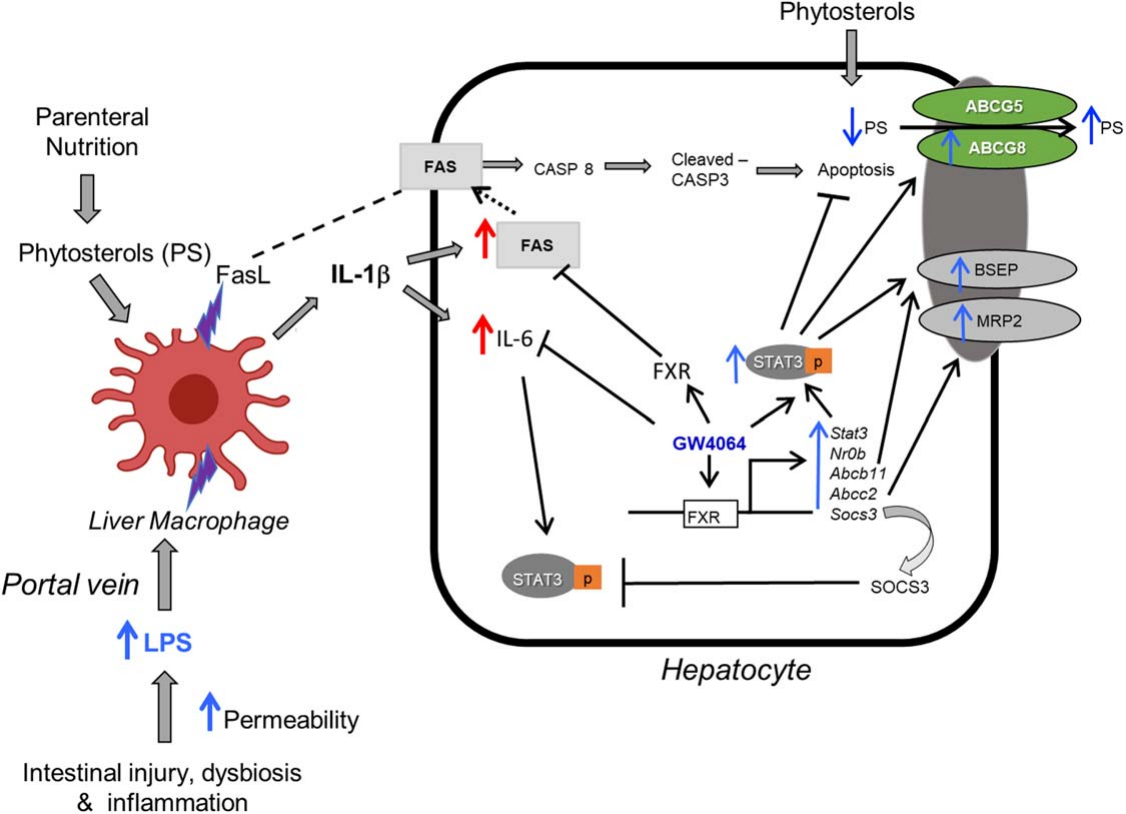
Proposed role of STAT3 in mediating GW4064 protection in parenteral nutrition-associated cholestasis. Intestinal injury, dysbiosis, and hyperpermeability caused by intestinal failure promote LPS absorption (and other pathogen associated molecular patterns) into portal vein, subsequently recruiting and activating liver macrophages to produce FAS ligand (CD178) and IL-1β, which promote hepatocyte FAS-induced apoptosis and IL-6 generation, respectively. FXR agonist GW4064 amplifies the activation of hepatocyte STAT3 that is associated with multiple protective effects, including suppression of IL-6, inhibition of apoptosis, and upregulation of canalicular bile transporters. Upregulation of ABCG5/G8 promotes increased hepatocyte canalicular secretion of phytosterols resulting in lower hepatocyte concentrations and less antagonism of FXR signaling, culminating in improved bile secretion and prevention of cholestatic injury. Abbreviations: CASP-8, Caspase 8; FAS, Fas-associated protein with death domain; FXR, farnesoid X receptor; LPS, lipopolysaccharides; MRP2, multidrug resistance-associated protein 2; STAT3, signal transducer and activator of transcription 3.

In this study, we investigated the role of the STAT3 signaling pathway and its interaction with FXR gene regulation in the PNAC mouse model. In this model, intestinal injury and increased intestinal permeability to LPS derived from intestinal microbiota in conjunction with infusion of soybean oil-lipid emulsion containing PN solutions mirrors the pathophysiology present in humans with PNAC and intestinal failure-associated liver disease.^4^,^6^,^7^,^9^,^10^,^23^,^36^ We have previously demonstrated in this model the importance of the hepatic inflammatory environment (increased gene expression of *Il-1b*, *Tnf*, and *Il-6*), hepatic macrophage infiltration and activation, hepatic retention of PS, suppression of FXR signaling, and hepatic bile and sterol gene transporter downregulation in the pathogenesis of PNAC; similar findings have been reported by others in children with intestinal failure-associated liver disease.^4^,^9^,^10^,^23^,^36^ In the current study, we identify that hepatic *Stat3* is upregulated and activated by IL-1β signaling in the PNAC mouse, but this seemed insufficient to ameliorate the cholestasis and hepatocyte apoptosis. However, treatment with the FXR agonist GW4064 induced increased FXR binding to the *Stat3* promoter and further upregulation of STAT3 signaling, which protected against hepatocyte apoptosis and PNAC.^6^,^7^ These findings were supported in IL-1 receptor-deficient/DSS-PN mice and cultured cells exposed to IL-1β, PS, and GW4064. Thus, this study proposes a novel mechanism by which FXR agonists may protect and reverse cholestasis in PNAC through activation of STAT3 signaling (Figure 7).

It has been proposed that STAT3 plays an important role in the suppression of hepatic injury during cholestasis.^45^ In the present study, treatment with an FXR agonist led to increased FXR binding to the *Stat3* promoter in PNAC mouse liver, inducing STAT3 phosphorylation associated with enhanced expression of FXR target genes. However, the upregulation by FXR on its target genes (*NROB2*, *ABCB11* and *ABCG8*) in the presence of IL-1β was largely prevented when STAT3 was knocked down by siRNA in cultured cells (Figure 5). Moreover, when STAT3 was knocked down, PS-mediated inhibition of FXR target gene expression was magnified (Figure 6), likely because of the reduced expression of the sterol exporter *ABCG8* resulting in increased hepatocyte concentrations of PS. These data strongly suggest that the effect of the FXR agonist in increasing expression of canalicular transporters for bile acids and PS and in reducing hepatocellular apoptosis, was mediated, at least in part, through STAT3 signaling.

In our previous report, we showed that in PNAC, hepatic macrophage-derived IL-1β induced activation of NFκB in hepatocytes where it binds to promoter sequences and interferes with expression of FXR regulated bile-transporter and sterol-transporter genes.^6^,^36^ In this study, we extend these findings by showing that macrophage-derived IL-1β induced the activation of apoptotic pathways in hepatocytes.^46^,^47^ LPS-treated mice had increased the production of FasL/CD178 in purified F4/80-positive hepatic macrophages,^6^,^36^ and IL-1β-injected mice had upregulation of *Fas*/CD95. Moreover, FAS and caspase 8 were upregulated in livers of PNAC mice, which were normalized in *Il1r* ^*−/−*^/DSS-PN mice or in DSS-PN mice treated with the FXR agonist. We further show that siRNA knockdown of *STAT3* in IL-1β-treated HepG2 cells led to decreased *ABCB11*, *NR0B2*, and *ABCG8* mRNA expression, even in the presence of the FXR agonist, indicating an effect of STAT3 on the interaction between FXR and its target genes.^48^ Thus, IL-1β, which is activated in liver macrophages of PNAC mice, induces both apoptotic hepatocyte pathways and suppression of FXR target canalicular transporters in PNAC liver, all of which are reversed by induction of STAT3 signaling by the FXR agonist.

Previous reports support a role for STAT3 signaling in cholestasis. *Stat3* ^*−/−*^ hepatocytes are more susceptible to bile acid–induced injury^45^ and liver injury in *Mdr*2^*−*/*−*^ mice was exacerbated by *STAT3* inactivation. In addition, *Stat3* ^−/−^ mice at baseline show increased hepatic infiltration of neutrophils and monocytes compared with WT mice, placing them at potentially increased risk for liver injury induced by innate immune activation, such as that observed in PNAC.^49^,^50^ In our experiments, we used siRNA knockdown of STAT3 in HepG2 and Huh7 cells to demonstrate the essential role of STAT3 signaling in maintaining expression of bile acid and sterol transporters in IL-1β and PS exposed hepatocyte-like cells. We did not use Stat3^*−*/*−*^ mice in our experiments, which would have been another design to demonstrate this effect of Stat3 signaling, which can be viewed as a limitation of our study.

We have previously established that PN soy oil–based lipid emulsions are involved in the pathogenesis of PNAC.^2^,^36^ PSs, present in PN lipid emulsions, antagonize FXR signaling and expression of hepatocyte *Abcb11* and *Abcc2* and can themselves activate hepatic macrophages.^7^,^23^ FXR is the dominant nuclear hormone receptor regulating bile acid synthesis and transporters.^7^ Previously, we reported that downregulation of *ABCG5/8* in hepatocytes in PNAC mice was associated with the retention of PS and reduced expression of canalicular bile transporters, but in the presence of the FXR agonist,^6^ these findings were reversed and cholestasis was prevented. We now show that upregulation of STAT3 signaling is essential for expression of the protective effects of the FXR agonist in the PNAC mouse.

In conclusion, in the current study, we demonstrate an important role for STAT3 signaling in the pathogenesis of PNAC. STAT3 signaling seemed to mediate, in part, the protective effects of the FXR agonist GW4064 in hepatocytes exposed to either IL-1β or PS, 2 factors critical in PNAC pathogenesis, and in the PNAC mouse model treated with GW4064. This study supports the proposed mechanism (Figure 7) of PNAC in which proinflammatory activation of hepatic macrophages is promoted by the mutual effect of intestinal-derived LPS and circulating PS,^23^,^36^ that, in turn, leads to downstream events in the hepatocyte causing cholestatic injury and apoptosis. The protective effect of FXR agonists in cholestasis may be mediated in part through stimulating STAT3 signaling.

## Supplementary Material
