# Supplementary figures and images for: Stat3 role in the protective effect of FXR Agonist in parenteral nutrition-associated cholestasis
Source: Hepatol Commun. 2023 Feb 27;7(3):e0056. doi: 10.1097/HC9.0000000000000056 (PMC9974070; doi:10.1097/HC9.0000000000000056)

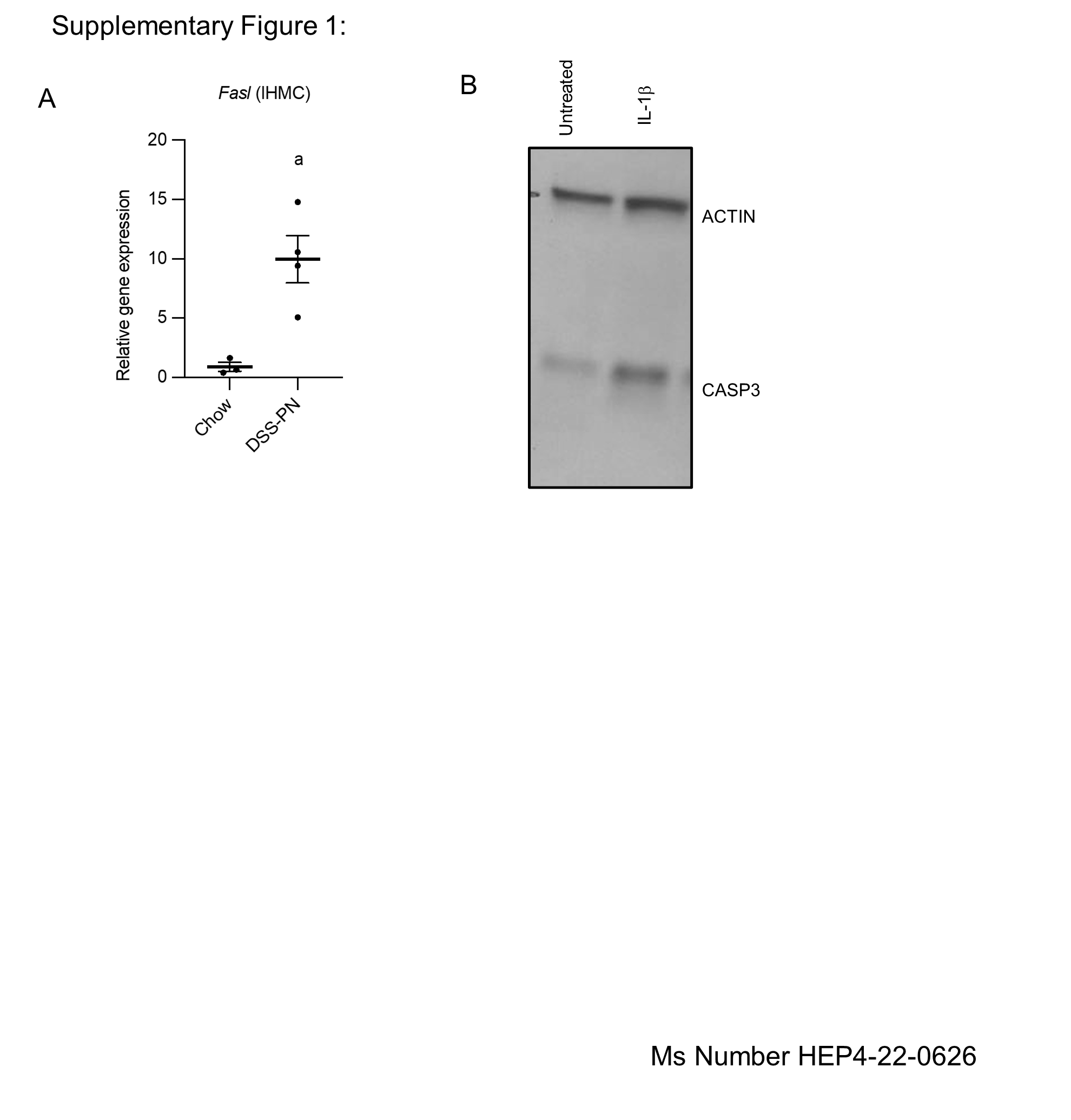

Supplement: Supplementary file 1 [file hc9-7-e0056-s001.tif]

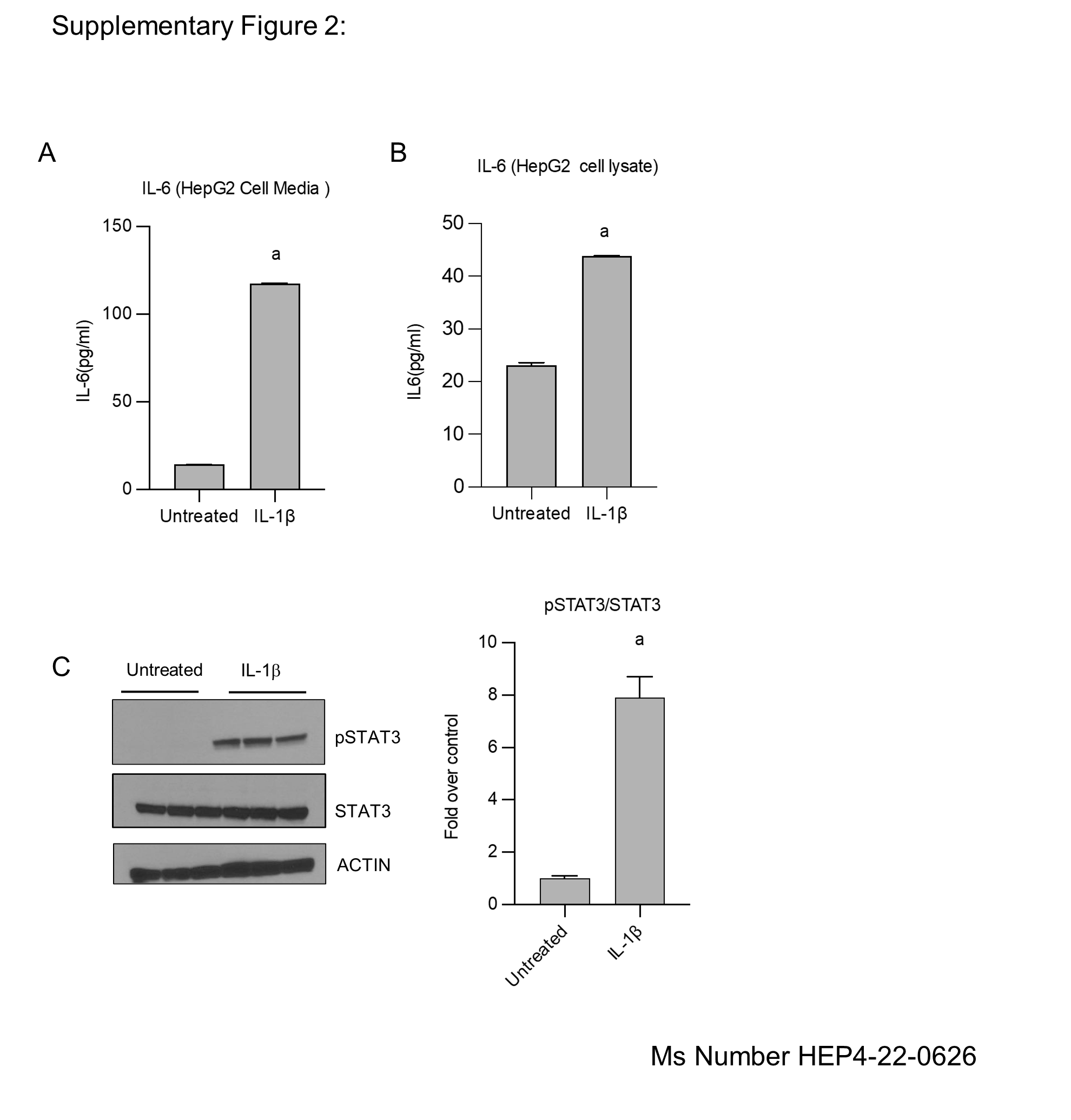

Supplement: Supplementary file 2 [file hc9-7-e0056-s002.tif]

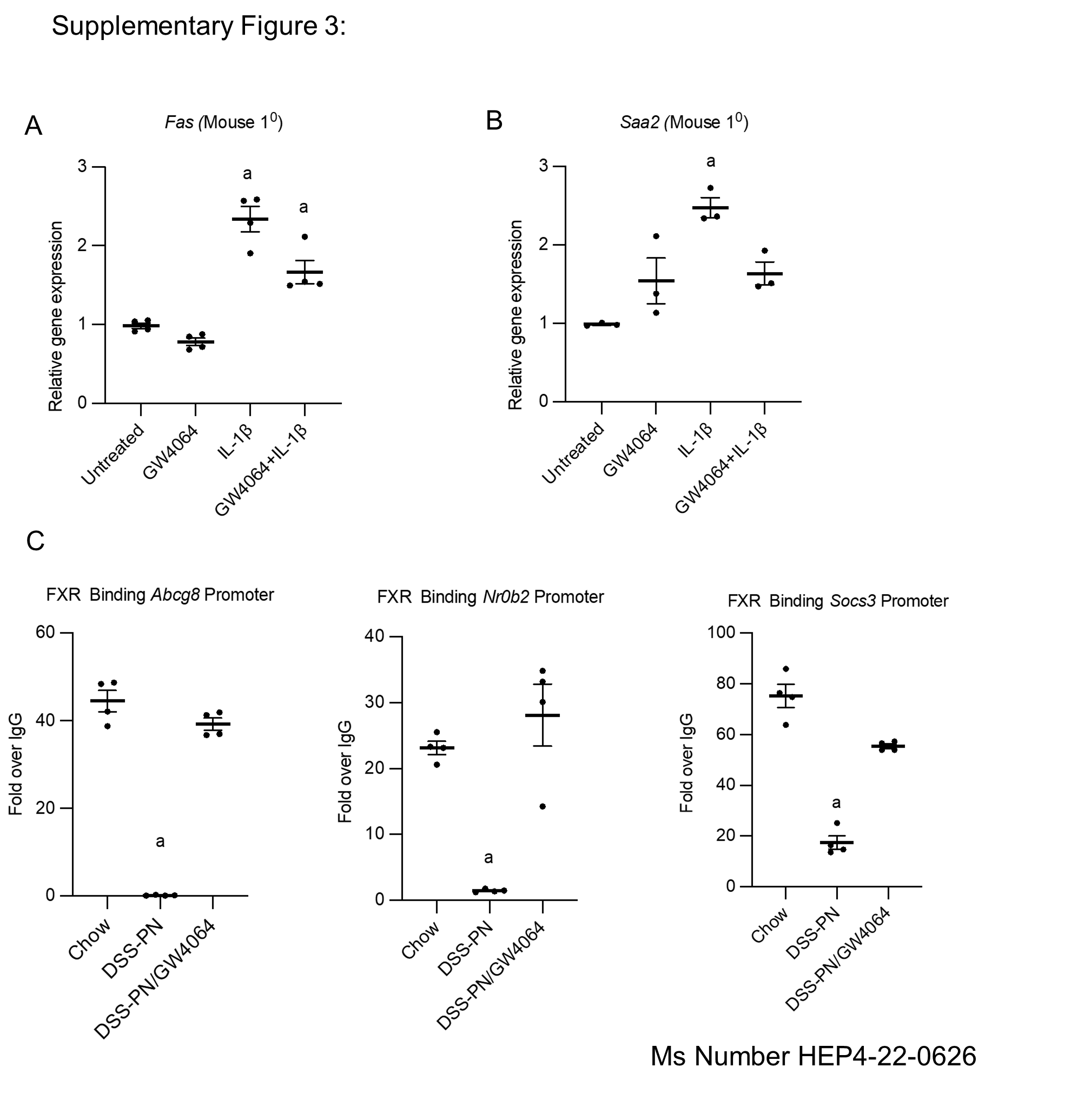

Supplement: Supplementary file 3 [file hc9-7-e0056-s003.tif]

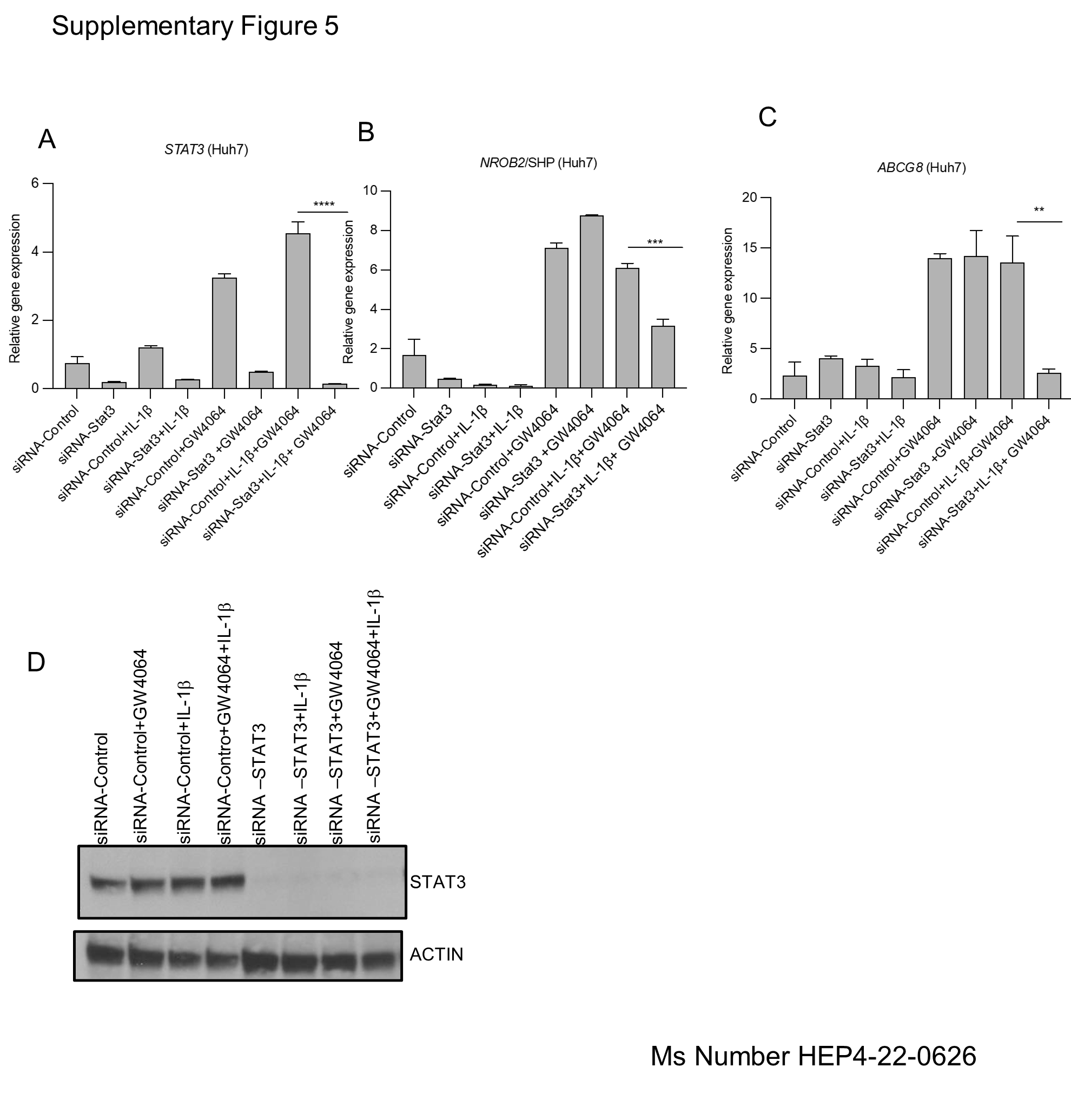

Supplement: Supplementary file 4 [file hc9-7-e0056-s004.tif]

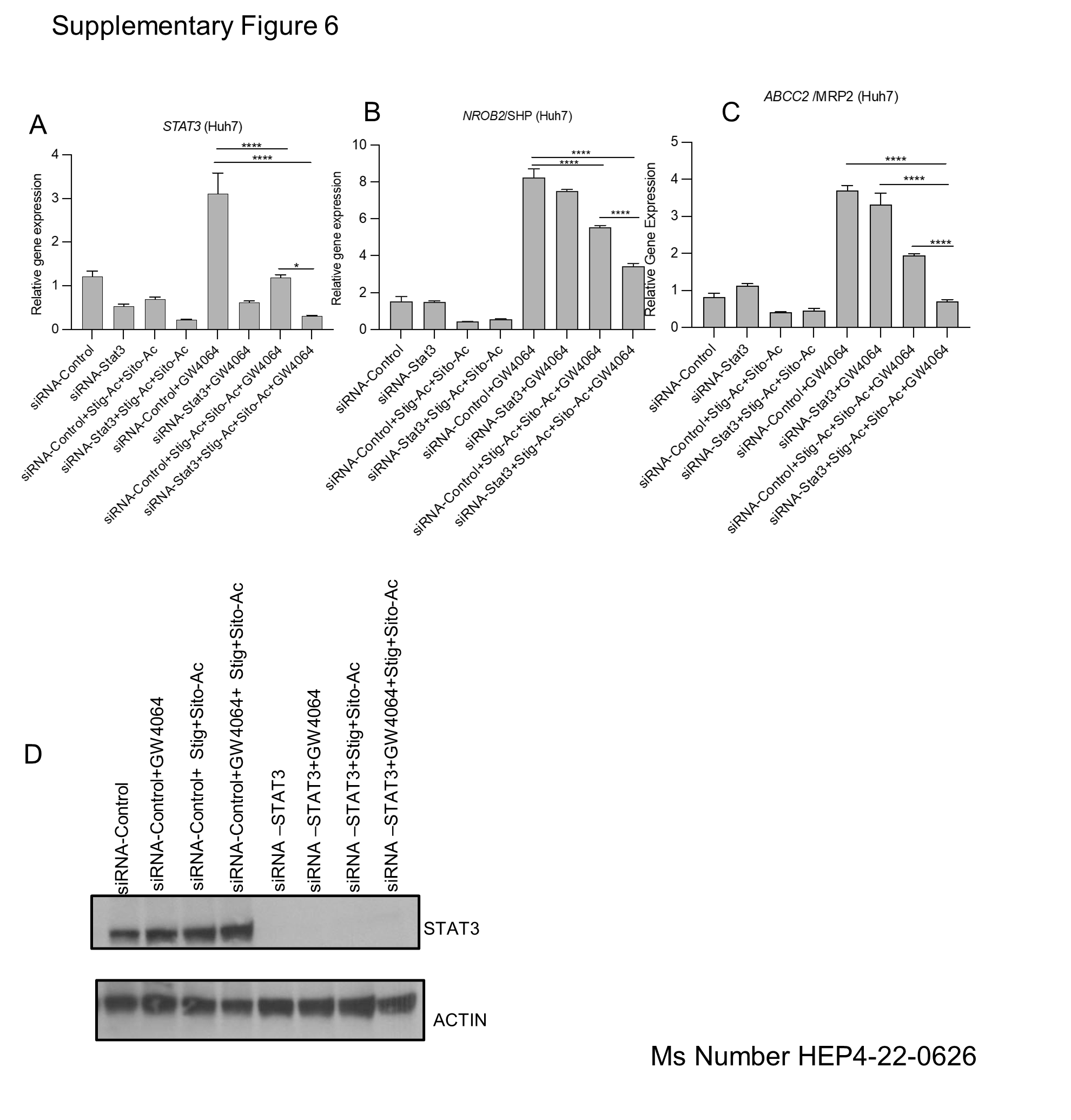

Supplement: Supplementary file 5 [file hc9-7-e0056-s005.tif]
