# Supplementary material for: Stat3 role in the protective effect of FXR Agonist in parenteral nutrition-associated cholestasis
Source: Hepatol Commun. 2023 Feb 27;7(3):e0056. doi: 10.1097/HC9.0000000000000056 (PMC9974070; doi:10.1097/HC9.0000000000000056)
Supplement: Supplementary file 6 [file hc9-7-e0056-s006.docx]

**Ms Number: HEP4-22-0626**

**SUPPLEMENTARY EXPERIMENTAL PROCEDURES**

**PNAC mouse model**

All animal procedures were approved by University of Colorado Anschutz Medical Campus Institutional Animal Care and Use Committee and all animals were treated humanitarianly during the experimental techniques, as earlier described in the PNAC mouse model (8,9,10,11,44). For the PNAC mouse model (8,9,10,11, 44) we used adult male C57BL/6 WT mice (8 weeks old, 22–23 g body weight) (Jackson Laboratories, Bar Harbor, ME, USA) that were maintained on a 12 h light/dark cycle and were boarded in specific pathogen-free conditions in individual metabolic cages. To produce a mild intestinal injury and altered intestinal barrier function with increased intestinal permeability, mice were exposed ad libitum to 2.0% dextran sulfate sodium (DSS; MP Biomedicals, Santa Ana, CA, USA) in the drinking water for 4 days whilst they had free access to chow and water ad libitum (referred to as “DSS pretreatment” period). Following the DSS pretreatment period, PNAC mice had placement of a central venous catheter (CVC) (Silastic tubing, 0.012 inches internal diameter; Dow Corning, IL, USA) while under pentobarbital anesthesia; the CVC was inserted into the right jugular vein and the proximal end was tunneled subcutaneously to exit between the shoulder plates. Mice were then placed in a rubber mouse harness (Instech Laboratories, Plymouth Meeting, PA, USA) and the CVC was threaded through the harness into a swivel apparatus and finally connected to an infusion pump (Harvard Apparatus, Holliston, MA, USA). Following recovery from anesthesia on a heating pad under continuous observation, mice were then observed for 24 h while receiving intravenous (i.v.) normal saline (NS) infusion at a rate of 0.23 ml/h and given ad libitum access to water and chow. Chow was removed after 24 h but free access to water was continued and PN was initiated as a continuous infusion for 14 days (DSS-PN mice) at a rate of 0.29 ml/h. This infusion rate provided a caloric intake of 8.4 kcal/day and included 20% soybean oil lipid emulsion (Nutralipid, Braun Medical Inc., Bethlehem, PA) at dose of 5 g/kg/day (7,12). Mice were randomly allocated to several additional experimental groups. Chow fed control mice (referred to as Chow mice) had free access to chow and water for a period of 19 days. In some experiments DSS-PN treatment was given to *Il1r−/−*mice. In another group, DSS pretreated mice were administrated PN containing the FXR agonist, (GW4064; Cat No 2473 ; Tocris, Minneapolis, MN, USA) dissolved in ethanol, at dose of 30 mg/kg/body weight per day(20) during day 4 through 14 of PN infusion. On the day of sacrifice, mice were anesthetized with i.p. pentobarbital and 100–400 µl of blood was collected from the retro-orbital plexus and liver was detached and divided into several pieces. One was placed in formalin for 4 h followed by placement in 70% alcohol. Another piece of liver was snap frozen in liquid nitrogen, coded, and subsequently stored at −80 °C until analyzed. From all mice similar lobes of liver were placed in formalin and similar lobes were snap frozen to ensure comparability and reproducibility. Coded serum samples were analyzed by the University of Colorado Hospital Clinical Chemistry Laboratory for AST and ALT (12). Total serum bile acids were analyzed in coded specimens using a Total Bile Acids Test Kit (Diazyme Laboratories, Poway, CA, USA; Cat No; DZ042A-KY1) and total serum bilirubin by a Bilirubin Colorimetric Assay Kit (Biovision Inc, Milpitas,CA; Cat No K553), according to the manufacturer’s instructions (7,12).

**Primary mouse hepatocyte isolation and culture**

Hepatocytes were isolated from fresh liver tissue as previously described (10,11). Briefly, a 24G catheter was placed into the portal vein and the mouse liver perfusion was done using sequential buffers as follows: 1) EGTA containing EBSS (Cat No: 14155 Gibco, Lafayette, CO, USA), 2) Liver perfusion media for 6 minutes (Cat No: 17701 Gibco), and 3) Liver digest media containing collagenase for 15 minutes (Cat No: 17703 Gibco) at 37oC. After perfusion, liver tissue was detached and filtered using 70 micron filter strainer and washed with William E media (Cat No: 32551, Gibco,USA). Following centrifugation at 25G for 5 minutes, the hepatocytes were resuspended in Williams E media.The cells were plated at a density of next morning, the primary mouse hepatocytes in culture were treated with 10 ng/ml IL-1β (BD Biosciences, San Jose, CA, USA) for 4h followed by 5 mM GW4064 overnight in William’s E media. Following day, cells were collected and used for RNA analysis.

**Supplementary Table 1. List of TaqMan Probes**

**Mouse:**

| Gene | Probe Set |
| --- | --- |
| *Fas* | Mm01204974 |
| *Saa2* | Mm04208126 |
| *Il-6* | Mm00446190 |
| *Fasl* | Mm00438864 |
| *Socs1* | Mm00782550 |
| *Socs3* | Mm00545913 |

**Human:**

| Gene | Probe Set |
| --- | --- |
| *STAT3* | Hs00374280 |
| *NR0B2* | Hs00222677 |
| *SOCS3* | Hs00223686 |
| *ABCG8* | Hs00223690 |
| *ABCB11* | Hs00994811 |
| *ABCC2* | Hs00960489 |
| *IL-6* | Hs00985639 |

**Supplementary Table 2. List of Antibodies**

| Antibody | Catalog No | Dilution | Vendor |
| --- | --- | --- | --- |
| STAT3 | 4904s | 1:1000 | Cell Signaling |
| Phospho STAT3 | 9145S | 1:1000 | Cell Signaling |
| FXR | 252165 | 1:1000 | Abbiotec |
| CASPASE 8 | 4927 | 1:1000 | Cell Signaling |
| Cleaved CASPASE 3 | 9661S | 1:1000 | Cell Signaling |
| FAS | Sc-8009 | 1:1000 | Santa Cruz Biotechnology |
| CD95 | 17-0951-8 | 1:200 | ThermoFisher Scientific |
| CD178 | 62-5911-82 | 1:200 | ThermoFisher Scientific |
| GRB2 | 3972 | 1:1000 | Cell Signaling |
| Actin | 12620S | 1:1000 | Cell Signaling |
| Anti-Mouse- IgG-HRP | HAF007 | 1:5000 | R&D systems |
| Anti-Rabbit -IgG-HRP | 3972 | 1:5000 | Santa Cruz Biotechnology |

**FIGURE LEGENDS FOR SUPPLEMENTAL FIGURES:**

# **Supplemental Figure 1**

**FAS mediated hepatic apoptosis is activated in the PNAC mouse.**

**(A)** qPCR of *Fasl* in purified IHMC from Chow and DSS-PN treated mice using *Hprt1* was as reference control and expressed relative to Chow controls. a=significantly different from Chow (p < 0.05). (**B)** Immunoblot analysis of Caspase-3 in mouse primary hepatocytes cells incubated with IL-1b/overnight.

Data indicate the mean ± SEM. a*p <* 0.05 by 2-tailed, unpaired Student’s *t* test . a=significantly different from all control groups, p <0.05 by one-way ANOVA.

# **Supplemental Figure 2**

**IL-1b promotes IL-6 secretion and STAT3 activation in HepG2 cells.**

HepG2 cells were incubated with IL-1b overnight after which cells were harvested and ELISA of *IL-6* performed. **(A)** HepG2 Cell media **(B)** HepG2 Cell lysate. (**C).** Immunoblot of pSTAT3 from HepG2 cells. a=significantly different from untreated (p <0.05) by 2-tailed, unpaired Student’s *t* test ‘

# **Supplemental Figure 3**

**GW4064 decreases IL-1b -induction of *Fas* and *Saa2.***

Cultured mouse primary hepatocytes (Mouse 1o)were incubated with IL-1b for 4h followed by GW4064 overnight after which cells were harvested and mRNA analysis performed for **(A)** *Fas* and **(B)** *Saa2***. (C)** ChIP assay for FXR binding to the promoter of *Abcg8, Nr0b2 and Socs3* inliver homogenate from Chow, DSS-PN and DSS-PN/GW4064 mice. Data presented as fold change over IgG.

a=significantly different from all other groups, p <0.05 by one-way ANOVA.

# **Supplemental Figure 4**

**GW4064 inhibits the IL-1β upregulation of IL-6 in Huh7 cells.**

**(A)** Huh7 cells (n=3) were incubated with IL-1b for 4h followed by GW4064 overnight after which cells were harvested and *IL-6* qPCR analysis was performed.

# **Supplemental Figure 5**

**STAT3 inhibition downregulates the expression of FXR target genes in Huh7 cells and HepG2 cells.**

Huh7 cells were transfected with *STAT3* siRNA or non-targeting siRNA control for 24h followed by addition of IL-1b and GW4064 overnight after which cells were harvested and qPCR analysis was performed. mRNA levels are shown for **(A)** *STAT3,* **(B)** *ABCG8* **(C)** and *NR0B2*. Data indicate the mean ± SEM of 3 technical replicates of a representative experiment that was repeated three times. *p *<* 0.05 by 1-way ANOVA; *p<0.05, **p< 0.01, ****p< 0.0001. **(D)** Immunoblot analysis of STAT3 protein in HepG2 cells*.*

# **Supplemental Figure 6**

**Down-regulation of *STAT3* increases inhibitory effect of PSs on FXR target gene in Huh7 cells and HepG2.**

Huh7 cells were incubated with *STAT3* for 24h followed by addition of +/- GW4064 or +/- stig+sito overnight, cells were harvested, and mRNA analyzed. (A) *STAT3,* (B) *NR0B2*, (C) *ABCC2/MRP2*. Data indicate the mean ± SEM of 3 technical replicates of a representative experiment that was repeated three times. *p *<* 0.05 by 1-way ANOVA; (D*)* Immunoblotting analysis of STAT3 from HepG2 cells.

1. El Kasmi KC, Anderson AL, Devereaux MW, Fillon SA, Harris JK, Lovell MA, et al. Toll-like receptor 4-dependent Kupffer cell activation and liver injury in a novel mouse model of parenteral nutrition and intestinal injury. *Hepatology.* 2012;55(5):1518-28.

2. El Kasmi KC, Vue PM, Anderson AL, Devereaux MW, Ghosh S, Balasubramaniyan N, et al. Macrophage-derived IL-1beta/NF-kappaB signaling mediates parenteral nutrition-associated cholestasis. *Nat Commun.* 2018;9(1):1393.

3. El Kasmi KC, Ghosh S, Anderson AL, Devereaux MW, Balasubramaniyan N, D'Alessandro A, et al. Pharmacologic Activation of Hepatic Farnesoid X Receptor Prevents Parenteral Nutrition Associated Cholestasis in Mice. *Hepatology.* 2021.
